# Increased Stromal Infiltrating Lymphocytes Are Associated with the Risk of Disease Progression in Mesenchymal Circulating Tumor Cell-Positive Primary Breast Cancer Patients

**DOI:** 10.3390/ijms21249460

**Published:** 2020-12-12

**Authors:** Bozena Smolkova, Zuzana Cierna, Katarina Kalavska, Svetlana Miklikova, Jana Plava, Gabriel Minarik, Tatiana Sedlackova, Dana Cholujova, Paulina Gronesova, Marina Cihova, Karolina Majerova, Marian Karaba, Juraj Benca, Daniel Pindak, Jozef Mardiak, Michal Mego

**Affiliations:** 1 Department of Molecular Oncology, Cancer Research Institute, Biomedical Research Center of the Slovak Academy of Sciences, Dubravska Cesta 9, 845 05 Bratislava, Slovakia; bozena.smolkova@savba.sk (B.S.); svetlana.miklikova@savba.sk (S.M.); jana.plava@savba.sk (J.P.); dana.cholujova@savba.sk (D.C.); paulina.gronesova@savba.sk (P.G.); marina.cihova@savba.sk (M.C.); kaja.majer@gmail.com (K.M.); 2 Department of Pathology, Faculty of Medicine, Comenius University, Sasinkova 4, 811 08 Bratislava, Slovakia; ciernaz@gmail.com; 3 Department of Pathology, Faculty Hospital, A. Zarnova 11, 917 75 Trnava, Slovakia; 4 2nd Department of Oncology, Faculty of Medicine, Comenius University and National Cancer Institute, Klenova 1, 833 10 Bratislava, Slovakia; katarina.kalavska@nou.sk (K.K.); jozef.mardiak@nou.sk (J.M.); 5 Translational Research Unit, Faculty of Medicine, Comenius University, Klenova 1, 833 10 Bratislava, Slovakia; 6 Department of Molecular Biology, Faculty of Natural Sciences, Comenius University in Bratislava, Ilkovicova 6, 842 15 Bratislava, Slovakia; gabriel.minarik@gmail.com; 7 Comenius University Science Park, Ilkovicova 8, 841 04 Bratislava, Slovakia; tatiana.sedlackova@gmail.com; 8 Geneton Ltd., Ilkovicova 8, 841 04 Bratislava, Slovakia; 9 Department of Oncosurgery, National Cancer Institute, Klenova 1, 83310 Bratislava, Slovakia; marian.karaba@nou.sk (M.K.); juraj.benca@nou.sk (J.B.); daniel.pindak@nou.sk (D.P.); 10 Department of Medicine, St. Elizabeth University, Namestie 1. maja 1, 811 02 Bratislava, Slovakia; 11 Department of Oncosurgery, Slovak Medical University, Limbova 12, 83103 Bratislava, Slovakia

**Keywords:** circulating tumor cells, tumor-infiltrating lymphocytes, primary breast cancer, cytokines

## Abstract

Circulating tumor cells (CTCs) and the immune infiltration of tumors are closely related to clinical outcomes. This study aimed to verify the influence of stromal lymphocyte infiltration and the immune context of tumor microenvironment on the hematogenous spread and prognosis of 282 chemotherapy naïve primary BC patients. To detect the presence of mesenchymal CTCs, RNA extracted from CD45-depleted peripheral blood was interrogated for the expression of mesenchymal gene transcripts. Tumor-infiltrating lymphocytes (TILs) were detected in the stromal areas by immunohistochemistry, using CD3, CD8, and CD45RO antibodies. The concentrations of 51 plasma cytokines were measured by multiplex bead arrays. TILs infiltration in mesenchymal CTC-positive patients significantly decreased their progression-free survival (HR = 4.88, 95% CI 2.30–10.37, *p* < 0.001 for CD3^high^; HR = 6.17, 95% CI 2.75–13.80, *p* < 0.001 for CD8^high^; HR = 6.93, 95% CI 2.86–16.81, *p* < 0.001 for CD45RO^high^). Moreover, the combination of elevated plasma concentrations of transforming growth factor beta-3 (cut-off 662 pg/mL), decreased monocyte chemotactic protein-3 (cut-off 52.5 pg/mL) and interleukin-15 (cut-off 17.1 pg/mL) significantly increased the risk of disease recurrence (HR = 4.838, 95% CI 2.048–11.427, *p* < 0.001). Our results suggest a strong impact of the immune tumor microenvironment on BC progression, especially through influencing the dissemination and survival of more aggressive, mesenchymal CTC subtypes.

## 1. Introduction

Breast cancer (BC) represents a heterogeneous disease that develops in a very complex microenvironment composed of several types of benign cells, some of which are involved in the immunogenicity of BC [1]. Although BC has not been considered as an “immunogenic“ malignancy, the occurrence of tumor-infiltrating lymphocytes (TILs) has been consistently documented, with an impact on prognosis [2]. TILs subpopulations are differently expressed in BC tumors, but their main component seems to be represented by CD3+ T-cells [3]. Higher levels of tumor-infiltrating effector T-cells are associated with better clinical outcomes in selected BC subtypes [4,5,6,7]. TILs are also a valuable predictive biomarker. BC patients with elevated TIL amounts had a significantly increased pathological complete response rate upon neoadjuvant chemotherapy compared to patients with poorly infiltrated tumors [7,8]. 

On the other hand, Liu et al. recently showed that increased stromal TILs are associated with circulating tumor cells (CTCs) and metastatic relapse in BC patients after neoadjuvant chemotherapy [9]. The metastatic process is responsible for around 90% of cancer-related deaths and remains the biggest challenge of the BC treatment. The presence of CTCs in peripheral blood is a negative prognostic marker for primary as well as metastatic BC [10,11,12,13,14]. The role of CTCs as an independent prognostic factor in progression-free survival (PFS) and overall survival (OS) was confirmed by several retrospective studies [15,16]. A critical step in the metastatic process is an activation of epithelial-to-mesenchymal transition (EMT) in tumor cells, leading to the expression of mesenchymal traits in epithelial cancer cells [17]. EMT results in several hybrid phenotypes, possessing both epithelial and mesenchymal features. Therefore, the epithelial cell adhesion molecule (EpCAM)-based methods, which have consistently confirmed the prognostic value of CTCs, can underestimate highly heterogeneous non-epithelial CTC sub-populations, frequently associated with poor prognosis [14,18,19,20,21]. CTCs can enter the blood circulation as single cells or as multicellular clusters composed of homotypic or heterotypic cells. Heterotypic CTC clusters incorporate stromal or immune cells together with cancer cells, which increases their likelihood to efficiently metastasize [22]. The role of neutrophils in enhancing the metastatic potential of CTC-neutrophil clusters as well as their role in the establishment of the metastatic niche was reviewed recently [23]. Due to their dual phenotype and the role in cancer biology, it was hypothesized that their function is dictated in a context-dependent fashion [24]. 

Among other features, the tumor microenvironment is responsible for immune cell recruitment [25]. However, the contribution of different TIL subpopulations to the biological and clinical tumor behavior remains unclear [26]. Immune cells move into tissues under the influence of specific cytokines, chemokines, and adhesion molecules that exert a high degree of complexity. They can directly stimulate immune effector and stromal cells and enhance anti-tumor immunity. On the contrary, cancer cells can also directly inhibit the immune cell function by decreasing cellular receptors expression or by suppressing the adaptive immune system response. The anti- or pro-tumor action of immune cells is amplified by cytokines released into circulation playing a role in increased invasiveness, progression, and prognosis of cancer. As shown recently, factors secreted by the tumor, stromal, or immune cells may affect the composition of the patient’s serum [27]. Therefore, circulating cytokine levels can become a non-invasive marker of immune derangement. 

Based on these and our previous findings, we hypothesize that the TILs location, in addition to their quantity, may contribute differently to BC outcomes with important consequences for their function and prognostic value. To confirm this hypothesis, we analyzed the influence of stromal lymphocyte infiltration and the immune context of tumor microenvironment on the hematogenous spread and prognosis of chemotherapy naïve primary BC patients. 

## 2. Results

Patients’ clinical characteristics are shown in Table 1. Most of the patients were older than 50 years (77%, *n* = 217), T-stage T1 (68.4%, *n* = 193), low or intermediate grade (62.8%, *n* = 174). The majority of them were diagnosed with invasive ductal carcinomas (88.6%, *n* = 247), out of which most were hormone receptor-positive (86.5%, *n* = 244) and HER-2/neu negative (85.8%, *n* = 242). The most frequent molecular subtype was luminal A (53.2%, *n* = 150), followed by luminal B (21.3%, *n* = 60) and HER2 positive (14.2%, *n* = 40) subtype. The triple-negative subtype represented the smallest group (11.3%, *n* = 32). More than half of the patients were LN negative (64.3%, *n* = 180) without lymphovascular invasion (76.4%, *n* = 175). The association of individual clinical characteristics with PFS is shown in Table S1. 

### 2.1. Association of TILs with Clinical Characteristics and Progression-Free Survival

**Figure 1:**
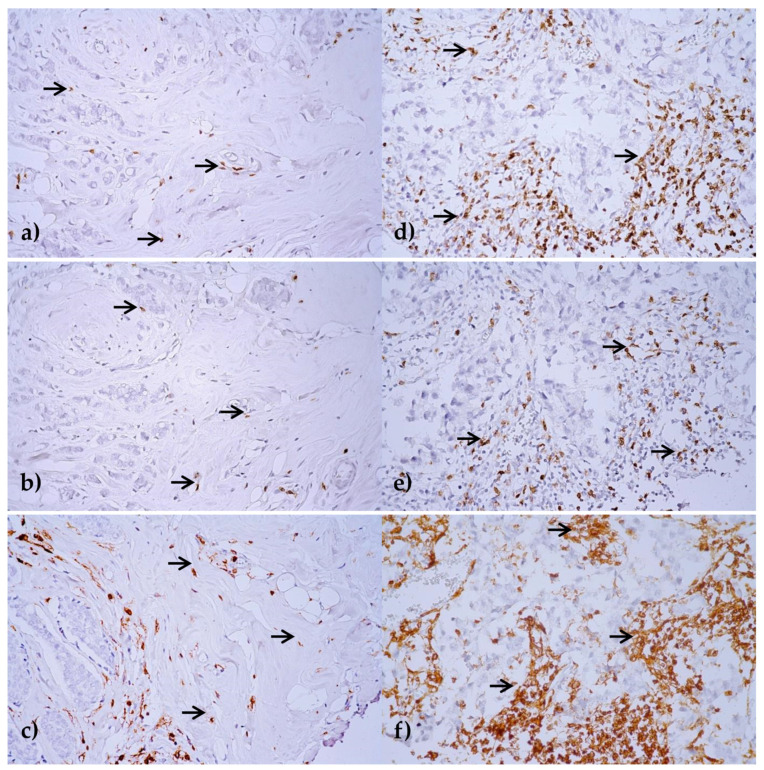
Immunohistochemical expression of CD3 (**a**,**d**), CD8 (**b**,**e**) and CD45RO (**c**,**f**) in stromal tumor-infiltrating lymphocytes (TILs). Breast cancer tissue areas with low (**a**–**c**), and high stromal TIL (**d**–**f**) infiltration are marked by arrows. Original magnification ×400, visualization with 3,3′-diaminobenzidine.

Stromal TILs, namely CD3, CD8, and CD45RO, were evaluated in the formalin-fixed paraffin-embedded (FFPE) tumor tissues by immunohistochemistry (IHC) as a percentage of area occupied by CD3, CD8, and CD45RO positive mononuclear inflammatory cells over the total intratumoral stromal area. Intratumoral TILs defined as lymphocytes in tumor nests directly interacting with tumor cells were not evaluated (Figure 1). 

Lymphocyte predominant tumors, defined using cut-off values of 60% and 50%, respectively, were rare. Based on CD3, it was 2.9% (*n* = 7) and 5.4% (*n* = 13) of tumors, 2.3% (*n* = 6) and 6.1% (*n* = 16) based on CD8, and 21.4% (*n* = 55) and 25.3% (*n* = 65) based on CD45RO TILs. 

The mean percent ± standard deviations (SD) for individual TILs were 13.0% ± 15.3 for CD3; 12.3% ±14.9 for CD8, and 30.5% ± 27.2 for CD45RO, while medians were 7% (ranging from 1–75%), 5% (ranging from 1–60%), and 20% (ranging from 1–90%), respectively. Individual TILs positively correlated with each other, CD3 and CD8, r = 0.773, *p* < 0.001; CD3 and CD45RO, r = 0.801, *p* < 0.001; CD8 and CD45RO, r = 0.681, *p* < 0.001. 

Based on the ROC curve analysis (Figure S1a–c), the values of CD3 above 6% were defined as CD3^high^, values below 6% as CD3^low^. Similarly, the values of CD8 above 6% were defined as CD8^high^, values below 6% as CD8^low^. Finally, the values of CD45RO above 12.5% were defined as CD45RO^high^, values below 12.5% as CD45RO^low^. Those values represent cut-offs for further analysis. 

The TILs mean values differed significantly across clinical categories (Table 1). A higher stromal infiltration was uniformly identified in patients with adverse outcomes, e.g., high grade, negative HR status, high Ki-67 proliferation index, and triple-negative tumor subtype. Less pronounced, but still significant differences were identified in IDC, HER2-, and bcl2-positive patients as well as those with LVI. 

**Figure 2:**
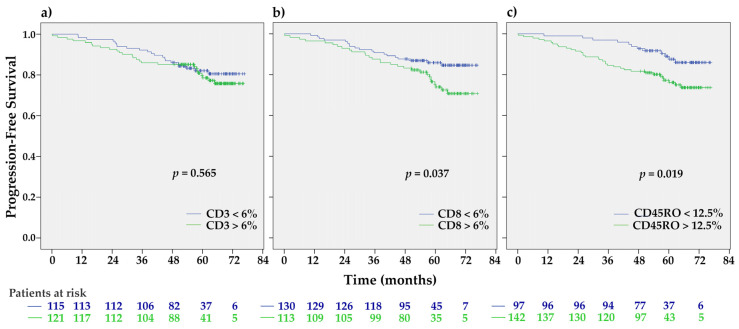
The Kaplan-Meier progression-free survival (PFS) estimates for studied stromal TILs expression. In contrast to patients with CD3 infiltration (**a**) those with CD8^high^ (**b**) and CD45RO^high^ (**c**) had significantly shorter PFS than those with CD8^low^ or CD45^low^, respectively (*p* = 0.037 and *p* = 0.019 by the Log-rank test).

High CD8 and CD45RO stromal infiltration were associated with shorter PFS (HR 1.83, 95% CI 1.03–3.27, *p* = 0.040, and HR 2.16, 1.12–4.17, *p* = 0.022, respectively) (Table S2, Figure 2). The subtype-specific prognostic significance of individual TILs is shown in Table S3. 

### 2.2. Association between TILs and CTCs and Their Prognostic Significance

**Figure 3:**
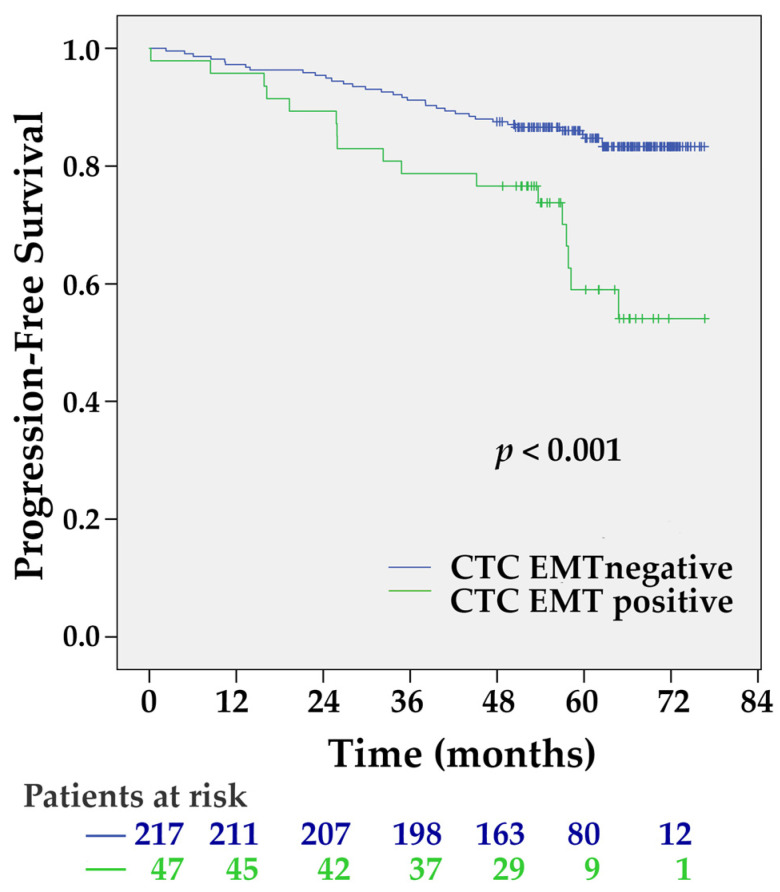
The Kaplan-Meier PFS estimates for circulating tumor cells (CTC) epithelial-to-mesenchymal transition (EMT). CTC EMT positive patients had significantly shorter PFS (*p* < 0.001 by the Log-rank test).

To determine the mRNA expression of EMT-inducing transcription factors (TF) in CD45 depleted fraction of peripheral blood, we compared the expression levels in patient samples with those of 60 healthy donors (Table S4). Among the patient samples, Twist1 and Slug transcripts were overexpressed in four (1.8%) and 44 (16.5%) samples, respectively (Table S5). Relative to the highest Snail and Zeb1 transcript levels detected in healthy donor samples, none of the patients overexpressed these gene transcripts. In total, CTC EMT positivity was detected in 17.6% (*n* = 47) of patients. The presence of CTC EMT was associated with decreased PFS (Figure 3) (HR 2.75, 95% CI 1.53–4.94, *p* = 0.001). 

**Figure 4:**
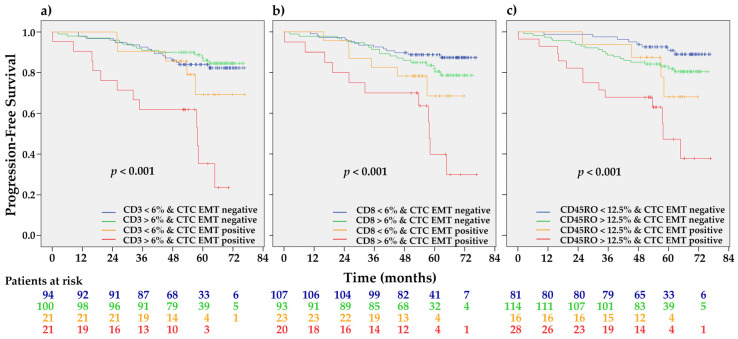
The Kaplan-Meier PFS estimates for CTC EMT stratified by individual stromal TILs categories. CTC EMT positive patients with (**a**) CD3^high^, (**b**) CD8^high^, and (**c**) CD45RO^high^ stromal infiltration had significantly shorter PFS than all other combinations (*p* ≤ 0.001 for CD3, CD8, and CR45RO, respectively, by the Log-rank test).

We did not find differences in stromal TILs infiltration between CTC EMT positive and negative patients (Table 1). CTC EMT-positive CD3^high^ (HR 4.88, 95% CI 2.30–10.37, *p* < 0.001), CD8^high^ (HR 6.17, 95% CI 2.75–13.80, *p* < 0.001), and CD45RO^high^ (HR 6.93, 95% CI 2.86–16.82, *p* < 0.001) patients had significantly shorter PFS in comparison with all other combinations (Figure 4).

### 2.3. Association between TIL Infiltration and Plasma Cytokine Levels 

**Figure 5:**
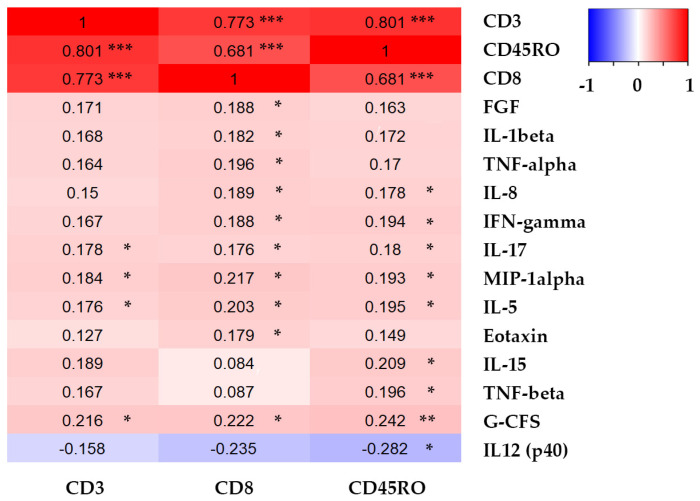
Heatmap showing correlation coefficients for significant correlations (highlighted by asterisks) found between CD3, CD8, and CD45RO TILs infiltration (%) and plasma concentrations of selected cytokines (pg/mL).

Cytokine measurement was done in the subgroup of 147 patients only. Although TILs strongly positively correlated with each other, correlations between plasma cytokine levels and TILs were rather weak, although several of them were significant (Figure 5). The most significant was the correlation between CD45RO and G-CFS (r = 0.242, *p* = 0.006). For all other identified correlations, the significance levels were close to the borderline value *p* < 0.05. All CD3, CD8, and CD45RO TILs correlated positively with IL-17, MIP-1α, IL-5, and G-CSF.

**Figure 6:**
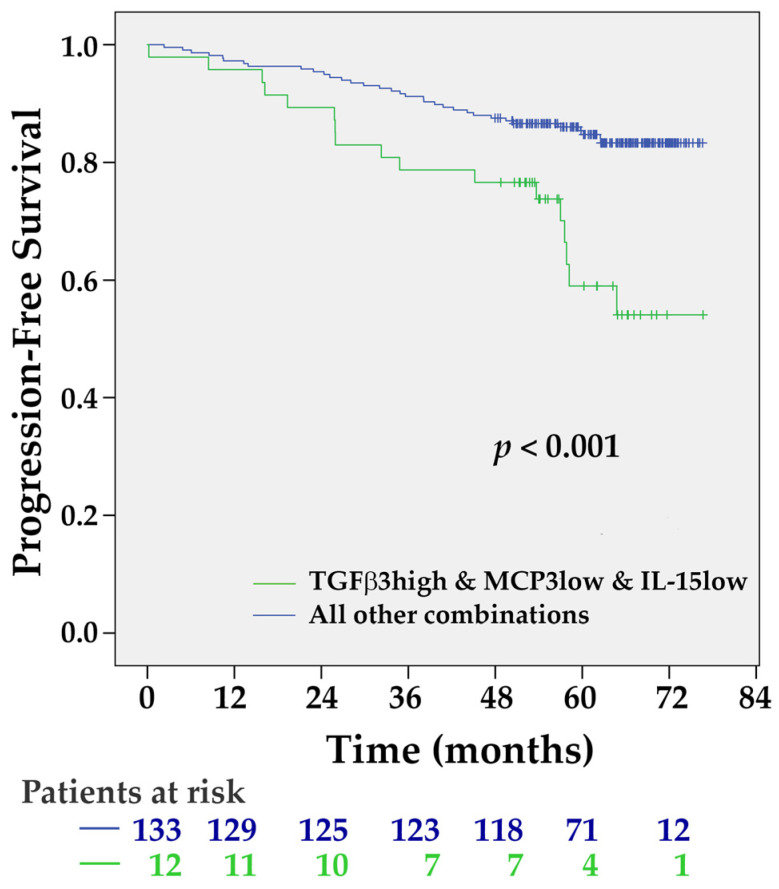
The Kaplan-Meier PFS estimates for the combination of TGF-β3^high^, MCP-3^low^, and IL-15^low^ plasma levels. Patients with this combination of plasma cytokine levels had shorter PFS, *p* < 0.001 by the Log-rank test.

All cytokines were dichotomized by the median values. The univariate Cox-proportional hazard model analysis was used for the selection of the most significant ones for multivariate regression analysis. Variables that achieved the *p*-value threshold *p* < 0.1 were included in the forward stepwise multivariate models. Three plasma cytokines, namely TGF-**β**3 (HR = 1.985, 95% CI 0.916–4.304, *p* = 0.082), MCP-3 (HR = 2.599, 95% CI 1.130–5.977, *p* = 0.025), and IL-15 (HR = 2.172, 95% CI 0.929–5.077, *p* = 0.073) fulfilled these criteria. Their combination (elevated plasma concentration of TGF-**β**3, cut-off 662 pg/mL marked as TGF-**β**3^high^, decreased MCP-3, cut-off 52.5 pg/mL marked as MCP-3^low^, and IL-15, cut-off 17.1 pg/mL marked as IL-15^low^) was significantly associated with an elevated risk of disease progression (HR = 4.838, 95% CI 2.048–11.427, *p* < 0.001) (Figure 6). 

### 2.4. Variables with the Most Significant Impact on Progression-Free Survival

The multivariate Cox logistic regression was employed to predict the recurrence probability in primary BC patients controlled for clinical predictor variables (listed in Table 1), stromal TILs infiltration, and cytokine levels. 

In addition to the known clinicopathological parameters, namely LN positivity and high Ki-67 proliferation index, plasma cytokines (TGF-β3^high^, MCP-3^low^, and IL-15^low^), and the interaction between stromal CD3, CD8, and CD45RO TILs infiltration and CTC EMT positivity were factors significantly associated with PFS (Table 2). 

## 3. Discussion

The prognostic role of lymphocytic infiltrates in BC was proposed in 1992 by Aaltomma et al. [28]. Since then, retrospective and prospective studies have shown that the presence of TILs is a predictive marker for higher responses to neoadjuvant chemotherapy and better survival, particularly in triple-negative and HER2-positive early BC [7,29]. Moreover, the presence of TILs in BC was identified as an independent predictor of the response to anthracycline/taxane neoadjuvant chemotherapy [30]. TILs were also shown to be an independent prognostic factor for disease-free survival, distant recurrence-free interval, and overall survival in triple-negative BC patients [4]. In the latter and ER+/HER2+ tumors, TILs have been associated with a significant reduction in the relative risk of death from the disease [5]. As shown above, the prognostic utility of TILs is related to the intrinsic subtypes and clinicopathological characteristics. Kurozumi et al. have shown high TILs expression to be a poor prognostic marker in ER-positive patients but a good prognostic marker in ER-negative patients [31]. Therefore, additional research should address the exact immune subsets of TILs and their prognostic utility in specific patient subgroups. 

Spatial TILs heterogeneity within the tumor can also influence their prognostic significance. Intratumoral TILs are defined as lymphocytes in direct cell-to-cell contact with carcinoma cells, while stromal TILs are located in the stroma between the carcinoma cells, but do not directly interact with them. TILs located intratumorally and those identified in stromal areas do not differ only in their contact with the cancer cells, but also in the degree of heterogeneity and the reaction to the signals from the microenvironment [32,33]. Importantly, the microenvironment consisting of a plethora of various non-cancerous cell types, extracellular matrix proteins as well as soluble molecules, can direct the fate of disseminated cancer cells, including cancer stem cells [33]. Based on the recommendations of an International TILs working group, the stromal TILs should be taken into account in the TILs evaluation [34]. The pooled data analysis of Loi et al. has shown the prognostic value of high stromal TILs in an early-stage, node-negative subgroup of triple-negative patients, who have low rates of recurrence and death [35]. Similarly, Rathore et al. have shown CD3+, CD4+, and CD8+ intratumoral and stromal TILs to predict favorable survival outcomes in infiltrating ductal carcinoma, where patients with intratumoral CD4+ and stromal CD8+ cells showed the highest survival [36]. Moreover, analyzing the patients treated with adjuvant anthracycline-based chemotherapy, Koletsa et al. have shown the correlation of higher stromal TILs density with a lower risk of relapse [37]. Oppositely, Liu et al. identified an increased stromal TILs and CD4+ T cell infiltration as an unfavorable prognostic factor measured by the rate of metastatic relapse [9]. To sum up, stromal TILs have an important prognostic and predictive value, especially in high-risk clinical BC subtypes. Recently, experts at the 16th St. Gallen Conference recommended their routine reporting in triple-negative patients [38]. Conflicting results published so far have been influenced by several factors, such as heterogeneity in lymphocyte distribution, technical slide-related issues, minimal assessable stroma, and other factors excellently discussed recently [39]. 

Our present data show the association of increased stromal TILs infiltration with shorter PFS, particularly in CTC EMT positive patients. A significant association between TILs and the presence of CTCs was demonstrated also in primary ovarian cancer patients [40]. In primary invasive BC, the prevalence of CTC was associated with an elevated number of intratumoral/peritumoral Tregs [41]. Increased infiltration of stromal TILs, CD4+, and CD8+ T cells after neoadjuvant chemotherapy was significantly correlated with the CTC presence [9]. There are no available published data assessing the prognostic value of stromal TIL infiltration in the context of hematogenous dissemination in chemotherapy naïve primary BC. To our best knowledge, our study is the first one concerning this association. 

Previously, we have shown the association between some plasma cytokines and their receptors with CTCs in peripheral blood of early BC patients [42]. In the present study, we assessed the same extensive number of cytokines out of which a combination of three, namely TGF-β3, MCP-3, and IL-15, was independently associated with an elevated risk of disease progression. The metastatic process is a complex phenomenon in which cytokines are crucial players. TGF-β, one of the EMT triggers, is well documented in several cancers, including BC [43,44,45]. Moreover, platelet-derived TGF-β was shown to activate TGF-β/Smad and NF-κ B pathways in tumor cells, thereby initiating and/or stabilizing their transition into an invasive mesenchymal-like phenotype [46]. MCP-3 (also known as CCL7) is a chemotactic factor and lymphocyte attractant, playing a pivotal role in tumorigenesis. It promotes EMT progression via the TGF-β pathway and facilitates tumor invasion and metastasis [47]. However, it was shown previously, that downregulated serum MCP3 levels in BC could reflect a lowered tumor immune surveillance by eosinophils, impaired maintenance of T-cell memory, and a reduced attraction of leucocyte subsets, which potentially recognize and destroy tumor cells [48]. As a pleiotropic cytokine, IL-15 plays an important role in innate and adaptive immunity and is able to activate the antimetastatic activities of NK cells by mediating the cross-talk with patrolling monocytes [49]. Decreased serum IL-15 levels can be a consequence of the attenuated antitumor response proposed by Jabri and Abadie [50]. Given the high prognostic significance of plasma concentrations of these three cytokines, we hypothesize that they can represent a surrogate marker of tumor immune derangement, driven by the influence of the tumor microenvironment and its immune infiltration. However, the limitation of our study is a relatively low number of peripheral blood samples for cytokine assessment. This is even more pronounced in the subgroup analysis. Therefore, we consider our findings for a combination of these plasma cytokine levels preliminary and propose they should be confirmed by other research groups. 

## 4. Materials and Methods 

### 4.1. Patients

In this translational study (Protocol TRU-SK 002; Chair: M.Mego), 282 primary BC patients with stages I–III, who were undergoing definitive surgery, were enrolled between March 2012 and February 2015. From each patient, peripheral blood for CTCs detection and cytokine assessment was obtained. The corresponding paraffin-embedded tumor tissues were collected for the TILs expression examination. Each patient was given a complete diagnostic evaluation to exclude the presence of distant metastasis. Patients with concurrent malignancy other than non-melanoma skin cancer in the previous 5 years were excluded as well. Clinicopathological data including information on age, tumor stage, histology, regional lymph node involvement, hormone receptor status, and HER2 status were also recorded. 

The study was approved by the Institutional Review Board (IRB) of the National Cancer Institute of Slovakia. Healthy donors (*N* = 60) were age-matched women without BC who were recruited and consented according to the IRB-approved protocol.

### 4.2. Detection of CTCs

CTCs were detected in peripheral blood by a quantitative real-time polymerase chain reaction (qRT-PCR) based assay utilizing CD45 positive (CD45+) cell depletion for CTCs enrichment, as described previously [51]. Peripheral blood was subjected to CD45 depletion using the RossetteSep^TM^ kit (Stem Cell Technologies, Vancouver, Canada) according to the manufacturer’s instructions. CD45-depleted cells were mixed with the TRIzolVR LS Reagent (Invitrogen Corporation, Carlsbad, CA, USA) and stored at −80 °C until RNA extraction. RNA from CD45-depleted cells was extracted with the TRIzolVR LS Reagent (Invitrogen Corporation, Carlsbad, CA, USA). The RNA concentration was determined by absorbance readings at 260 nm. RNA extracted from HeLa (ATCC^®^ CCL-2™), HCT 116 (ATCC^®^ CCL-247™), MCF7 (ATCC^®^ HTB-22™), and MDA-MB-231 (ATCC^®^ CRM-HTB-26™) cell lines were used as positive controls. 

Direct quantitative reverse transcription was used to detect EMT-inducing TF gene transcripts (*TWIST*, *SNAI1*, *SLUG*, and *ZEB1*). In brief, 1 μL of RNA was used in 20 μL of reaction volume containing 7.75 μL of water, 0.25 μL of One-Step RT-PCR enzyme mix, a combination of Omniscript and Sensiscript (both QIAGEN, Hilden, Germany) in the 1:1 ratio, 10 μL of Maxima Probe/ROX qPCR Master Mix (2X) (Thermo Fisher Scientific), and 1 μL of the assay. The following TaqMan assays were purchased from Life Technologies, USA: *TWIST1*: Hs00361186_m1; *SNAI1*: Hs00195591_m1; *SNAI2*: Hs00161904_m1; *ZEB1*: Hs01566408_m1; and *GAPDH:* Hs99999905_m1. Amplicons or probes spanned intron-exon boundaries. Amplification was performed on the Eppendorf Realplex Real-Time PCR system (Eppendorf, Germany) using the following cycling program: 50 °C for 30 min of reverse transcription, 95 °C for 10 min; 40 cycles of 95 °C for 15 s; and 60 °C for 60 s. Target cDNA was quantified using the delta-Ct method with the formula: 2 ^ (*C*t target—*C*t GAPDH).

Patient samples with higher EMT-associated TF gene transcripts than those of healthy donors were considered as CTC EMT positive. The highest expression levels of the EMT-inducing TF gene transcripts relative to that of GAPDH were 7.5 × 10^−4^, 3.8 × 10^−2^, and 1.7 × 10^−1^ for *TWIST1*, *SNAI1*, and *ZEB1*, while *SLUG* transcripts were not detected in any of the healthy donor samples. These values were used as a “cut-off” to determine CTC positivity (See Tables S4 and S5) [42].

### 4.3. Tumor Pathology

A pathology review was conducted at the Department of Pathology, Faculty of Medicine, Comenius University in Bratislava. The TILs assessment included tumor specimens from 271 patients. All specimens were classified according to the WHO Classification of 2012 [52]. 

According to the tumor histology, one or two representative tumor areas were identified in the hematoxylin and eosin sections. Sections were matched to their corresponding wax blocks (the donor blocks), and 3 mm diameter cores of the tumor were removed from these donor blocks with the multipurpose sampling tool Harris Uni-Core (Sigma-Aldrich, Steinheim, Germany) and inserted into the recipient master block. The recipient block was cut into 5-μm sections, and the sections were transferred to coated slides.

Slides were deparaffinized and rehydrated in a phosphate-buffered saline solution (10 mM, pH 7.2). The tissue epitopes were demasked using the automated water bath heating process in Dako PT Link (Dako, Glostrup, Denmark); the slides were incubated in a pH 6.0 citrate retrieval buffer at 98 °C for 20 min. The slides were subsequently incubated for 60 min at room temperature with the primary mouse monoclonal antibody against CD3 (Dako, M7254), CD8 (Dako, M7103), CD45RO (Dako, M0742) diluted 1:100 (CD3) or 1:200 (CD8, CD45RO). Immunostaining using anti-mouse/anti-rabbit immuno-peroxidase polymer (EnVision FLEX/HRP, Dako, Glostrup, Denmark) was performed for 30 min at room temperature. For visualization, the diaminobenzidine substrate-chromogen solution was used (DAB, Dako, Glostrup, Denmark) for 5 min. Finally, the slides were counterstained with hematoxylin (Sigma-Aldrich, Steinheim, Germany). For the negative control, breast tissue was subjected to the same procedure without staining with the primary antibody.

Tumor cores were independently assessed by a pathologist who was blinded to clinicopathological data. In cases of disagreement, the result was reached by consensus. The evaluation of TILs was performed according to the recommendations published by Salgado et al. [34]. In brief, we scored only stromal TILs as a percentage of the area occupied by CD3, CD8, and CD45RO positive mononuclear inflammatory cells over the total intratumoral stromal area. Intratumoral TILs defined as lymphocytes in tumor nests directly interacting with tumor cells were not evaluated. Altogether 248 samples were successfully scored for CD3, 270 for CD8, and 264 for CD45 expression. For better visualization, we used immunohistochemistry, similar to other published studies [5,30,53,54]. The percentage of positive stromal cells was assessed for all three markers.

### 4.4. Plasma Cytokines Assessment

In plasma samples of 147 patients, an analysis of 51 plasma cytokines and angiogenic factors was performed as published previously [42]. Human Group I and II cytokines and TGF-β panels were assessed using multiplex bead arrays (Bio-Plex 200 system, Bio-Rad Laboratories, Hercules, CA, USA). Human Group I 27-plex panel included the following targets: IL-1beta, IL-1r alpha, IL-2, IL-4, IL-5, IL-6, IL-7, IL-8, IL-9, IL-10, IL-12 (p70), IL-13, IL-15, IL-17, Basic FGF, Eotaxin, G-CSF, GM-CSF, IFN-gamma, IP-10, MCP-1 (MCAF), MIP-1alpha, MIP-1beta, PDGF-BB, RANTES, TNF-alpha, and VEGF (Bio-Plex Pro Human Cytokine 21-plex Immunoassay, Bio-Rad Laboratories, Hercules, CA, USA). Group II 21-plex, ICAM-1, and VCAM-1 panel contained targets: IL-1alpha, IL-2Ralpha, IL-3, IL-12 (p40), IL-16, IL-18, CTACK, GRO-alpha, HGF, IFN-alpha2, LIF, MCP-3, M-CSF, MIF, MIG, beta-NGF, SCF, SCGF-beta, SDF-1alpha, TNF-beta, and TRAIL, ICAM-1, and VCAM-1 (Bio-Plex Pro Human Cytokine 21-plex Immunoassay, Bio-Rad Laboratories, Hercules, CA, USA). TGF-β1, TGF-β2, and TGF-β3 were analyzed using the Bio-Plex Pro TGF-beta 3-plex Immunoassay (Bio-Rad Laboratories, Hercules, CA, USA). Premixed cytokine standards and samples were diluted following the manufacturer’s instructions and incubated with agitation (300 rpm, RT) with color-coded magnetic beads conjugated with monoclonal antibodies in the 96-well filter plate for 30 min (2 h for the TGF-β assay). As all three TGF-β isoforms are secreted as inactive complexes, samples were first activated with 1 N HCl for 10 min, then neutralized with 1.2 N NaOH/0.5M HEPES (Applichem, Darmstadt, Germany), and assayed immediately after the neutralization step. Following three washes, samples were incubated with a biotinylated detection antibody on a plate shaker (300 rpm agitation, RT) for 30 min in the dark (1 h for TGF-β). Each captured analyte was detected by the addition of streptavidin-phycoerythrin and quantified using a BioPlex suspension array reader (Bio-Rad Laboratories, Hercules, CA, USA) equipped with a 532 nm reporter laser and 635 nm classification laser diode. Cytokine concentrations (pg/mL) were calculated with the Bio-Plex Manager 4.0 software (Bio-Rad Lab, CA USA) using 5-parameter logistic (5PL) curve fitting. Medians of all measured cytokines are listed in Table S6.

### 4.5. Statistical Analysis

The patients’ characteristics were summarized using the median (range) for continuous variables and frequency (percentage) for categorical variables. The values of cytokines were dichotomized with the cut-off level of the median into two categories: Low (values below median) or high (values above the median). Normality of distribution was tested by the Kolmogorov-Smirnoff test. If normally distributed, sample means were tested by the Student *t*-test or the analysis of variance (ANOVA) with Bonferroni’s or Tamhane’s corrections, depending on the homogeneity of variance in the univariate analysis. For non-normally-distributed data, nonparametric Mann-Whitney U or the Kruskal-Wallis H test was used. Pearson’s or Spearman’s correlations were applied according to the normality of data. The univariate analyses were performed for categorical variables using χ^2^ or Fisher’s exact test. 

The receiver operator characteristic (ROC) analyses were applied to calculate the cut-off value for individual TILs, providing the highest sensitivity and specificity and to evaluate their prognostic accuracy. The cut-off values for clinical variables were chosen according to clinically significant values. 

The median follow-up period was calculated as a median observation time of all patients, as well as of those being still alive at the time of the last follow-up. PFS was calculated as the interval from the date of sampling (mostly date of surgery) to the date of progression, death, or date of the last adequate follow-up. PFS rates were estimated using the Kaplan-Meier product-limit method and the differences between survival curves were evaluated by the Log-rank test. 

Estimates of hazard ratios were calculated using the univariate Cox proportional hazard regression analysis. Factors affecting PFS were determined by the multivariate Cox proportional hazard model, applied to estimate the hazard ratio of each covariate and to adjust for potential confounders. Each model included age, clinicopathological characteristics (Table 1), a combination of cytokines preselected in the univariate analysis, and studied TILs expression. A backward model selection was conducted, and the final fitted model is presented. 

All presented *p*-values were two-sided and *p* < 0.05 was considered significant. Statistical analyses were performed using the IBM SPSS statistics version 23.0 software for Windows (IBM Corp. Released 2015. IBM SPSS Statistics for Windows, Version 23.0. Armonk, NY, USA: IBM Corp.)

## 5. Conclusions

In the present study, we evaluated the expression of stromal TILs, specifically T lymphocytes (CD3), cytotoxic T lymphocytes (CD8), and memory T lymphocytes (CD45RO) in breast tumor tissues and we correlated their expression in the stromal areas of tumors with the presence of CTC EMT in peripheral blood of patients. Previously, we have shown that abnormalities in T-cell-mediated immunity found in inflammatory CTC positive BC patients could potentially initiate and impact the dissemination of tumor cells [55]. Here, we have demonstrated the possible influence of stromal TILs infiltration on the hematogenous spread in primary BC patients. Moreover, we also interrogated changes in the plasma cytokine profile, which can serve as a surrogate marker of tumor-induced immune derangement. A combination of TGF- β^high^, MCP-3^low^, and IL-15^low^ at a given cut-off has the potential, after further validation on a bigger sample size, to serve as a non-invasive prognostic biomarker. 

## Figures and Tables

**Table 1:** Mean values of studied TILs stratified by clinicopathological characteristics.

Variables	Categories	*N*	%	CD3 Mean% ± SD	*p*	CD8 Mean% ± SD	*p*	CD45RO Mean% ± SD	*p*
All patients		282	100	13.00 ± 15.30		12.29 ± 14.89		30.48 ± 27.18	
Age (years)	≤50	65	23.0	15.38 ± 17.95	0.478	14.97 ± 17.71	0.405	34.28 ± 30.44	0.369
	>50	217	77.0	12.33 ± 14.46		11.45 ± 13.85		29.32 ± 26.08	
T-stage	T1	193	68.4	11.14 ± 13.16	0.101	11.81 ± 14.61	0.368	27.60 ± 24.60	0.086
	T2 and more	89	31.6	16.97 ± 18.58		13.29 ± 15.52		36.51 ± 31.22	
Histology	IDC	247	87.6	13.86 ± 15.87	0.020	13.18 ± 15.53	0.035	31.99 ± 27.66	0.016
	Others	35	12.4	6.46 ± 7.35		5.68 ± 5.39		18.62 ± 19.63	
Grade	Low and intermediate	174	62.8	9.08 ± 11.38	<0.001	10.13 ± 13.02	0.003	23.79 ± 22.66	<0.001
	High	103	37.2	19.88 ± 18.48		16.15 ± 17.10		42.05 ± 30.31	
N stage	N0	180	64.3	12.27 ± 15.46	0.124	11.80 ± 15.05	0.247	28.61 ± 27.33	0.046
	N+	100	35.7	14.31 ± 15.13		12.91 ± 14.68		33.97 ± 27.00	
LVI	Absent	175	76.4	11.91 ± 14.88	0.068	11.95 ± 14.89	0.024	28.05 ± 26.19	0.034
	Present	54	23.6	16.72 ± 17.27		14.94 ± 16.13		37.48 ± 29.43	
HR status ^$^	Negative	38	13.5	24.74 ± 19.34	<0.001	18.19 ± 16.66	0.005	54.54 ± 29.85	<0.001
	Positive	244	86.5	11.251 ± 13.82		11.31 ± 14.39		26.43 ± 24.55	
HER2 status	Negative	242	85.8	12.00 ± 14.80	0.018	11.69 ± 14.70	0.061	28.63 ± 26.31	0.013
	Amplified	40	14.2	18.43 ± 16.96		15.92 ± 15.72		41.49 ± 29.92	
p53	Negative	177	63.0	11.27 ± 13.69	0.124	11.60 ± 14.96	0.227	28.92 ± 26.39	0.281
	Positive	104	37.0	15.65 ± 17.35		13.52 ± 14.84		33.03 ± 28.52	
bcl2	Negative	80	28.4	17.85 ± 18.90	0.033	14.08 ± 15.46	0.133	39.67 ± 31.88	0.008
	Positive	202	71.6	11.10 ± 13.23		11.58 ± 14.65		26.90 ± 24.28	
Ki-67 ^&^	Low	180	63.8	8.94 ± 11.35	<0.001	10.36 ± 14.10	0.001	22.81 ± 21.67	<0.001
	High	102	36.2	19.83 ± 18.43		15.59 ± 15.70		43.56 ± 30.54	
Tumor subtypes	Luminal A	150	53.2	8.87 ± 10.90	<0.001	10.53 ± 14.13	0.006	22.08 ± 20.80	<0.001
	Luminal B	60	21.3	13.35 ± 16.48		10.51 ± 13.54		30.39 ± 26.80	
	HER2 positive	40	14.2	18.43 ± 16.96		15.92 ± 15.72		41.49 ± 29.92	
	Triple-negative	32	11.3	24.38 ± 20.43		18.66 ± 17.38		53.06 ± 31.64	
CTC EMT	Negative	220	82.4	12.98 ± 15.33	0.806	12.47 ± 15.20	0.774	29.69 ± 27.23	0.414
	Positive	47	17.6	13.07 ± 15.31		11.74 ± 14.60		31.55 ± 26.76	
Cytokines *	Low risk	133	91.7	12.54 ± 14.36	0.548	11.40 ± 13.58	0.056	27.77 ± 24.84	0.655
	High risk	12	8.3	13.60 ± 19.64		9.40 ± 18.73		27.30 ± 31.98	

The total number of samples analyzed in the study was *n* = 282; solely cases with valid information on individual variables were included in the table; ^$^ negative for both or positive for either with cut-off 1%; ^&^ cut-off 20%; * cut-off values: Transforming growth factor beta-3 (TGF-β3) > 662 pg/mL; monocyte chemotactic protein-3 (MCP-3) < 52.5 pg/mL and interleukin-15 (IL-15) < 17.1 pg/mL, data from two of 147 patients were not included, as they did not have successfully measured at least one cytokine used to set the cut-off value. Abbreviations: IDC: Invasive ductal carcinoma; LVI: Lymphovascular invasion; HR: Hormonal receptor.

**Table 2:** Cox proportional hazard regression models for the association between clinical predictor variables, plasma cytokines, the interaction between individual TILs and CTC EMT, and PFS.

Model	Variable	HR	95% CI	*p*
1	LN+	6.446	2.56–16.24	<0.001
	Ki-67 > 20%	12.00	4.22–34.11	<0.001
	Cytokines *	5.928	2.12–16.58	0.001
	CD3^high^ and CTC EMT positivity	5.277	2.09–13.30	<0.001
2	LN+	4.256	1.79–10.10	0.001
	Ki-67 > 20%	8.482	3.25–22.13	<0.001
	Cytokines *	6.387	2.35–17.38	<0.001
	CD8^high^ and CTC EMT positivity	3.655	1.49–9.00	0.005
3	LN+	3.796	1.50–9.58	0.005
	Ki-67 > 20%	7.251	2.59–20.30	<0.001
	Cytokines *	5.172	1.80–14.87	0.002
	CD45RO^high^ and CTC EMT positivity	4.922	1.83–13.23	0.002

Categorical variables entered in step 1: Age categories; T-stage; Histological grade; Ki-67, cut-off 20%; HER2 status; HR status, cut-off 1%; Grade; N-stage; * TGF-β3^high^, MCP-3^low^, and IL-15^low^; interaction of CD3, CD8, and CD45RO values with CTC EMT positivity.

## Supplementary Materials

The following are available online at https://www.mdpi.com/1422-0067/21/24/9460/s1, Figure S1: ROC curves for CD3, CD8, and CD45RO; Table S1: Univariate Cox logistic regression analysis for individual clinical variables; Table S2: Univariate Cox logistic regression analysis for individual studied TILs and PFS; Table S3: Univariate Cox logistic regression analysis for studied TILs, stratified by clinicopathological categories and PFS; Table S4: qRT-PCR results for control samples; Table S5: qRT-PCR results for patient samples; Table S6: Measured cytokines median levels (pg/mL).

## Abbreviations

BC	Breast cancer
CAF	Cancer-associated fibroblast
CTC	Circulating tumor cell
ECM	Extracellular matrix
EMT	Epithelial-mesenchymal transition
EpCAM	Epithelial cell adhesion molecule
Basic FGF	basic fibroblast growth factor
G-CSF	Granulocyte colony-stimulating factor
IDC	Invasive ductal carcinoma
IFN-gamma	Interferon gamma
IL	Interleukin
LVI	Lymphovascular invasion
MCP-3	Monocyte chemotactic protein 3
MDSC	Myeloid-derived suppressor cell
MET	Mesenchymal-epithelial transition
MIP-1alpha	Macrophage inflammatory protein-1α
OS	Overall survival
PFS	Progression-free survival
TAM	Tumor-associated macrophage
TGF-beta	Transforming growth factor-beta
TIL	Tumor-infiltrating lymphocyte
TNF-alpha	Tumor necrosis factor-alpha
TNF-beta	Tumor necrosis factor-beta
