# Supplementary material for: Increased Stromal Infiltrating Lymphocytes Are Associated with the Risk of Disease Progression in Mesenchymal Circulating Tumor Cell-Positive Primary Breast Cancer Patients
Source: Int J Mol Sci. 2020 Dec 12;21(24):9460. doi: 10.3390/ijms21249460 (PMC7763628; doi:10.3390/ijms21249460)
Supplement: Supplementary file 1 [file ijms-21-09460-s001.pdf]

Supplementary material

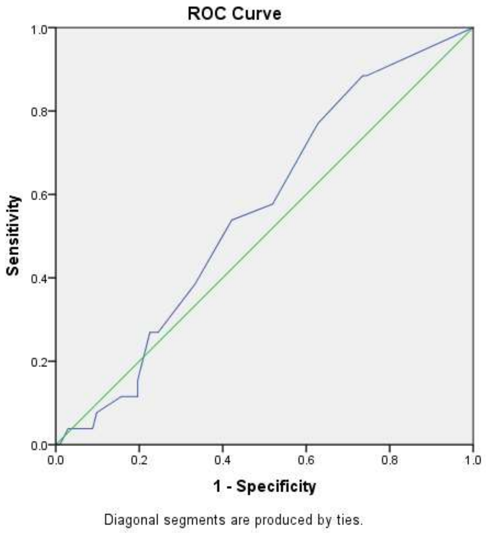

FigureS1a. ROC curve for CD3, AUC value is shown in the table below

Area Under the Curve

Test Result Variable(s): CD3

Area	Std. Error[a]	Asymptotic Sig.[b]	Asymptotic 95% Confidence Interval: Lower Bound	Asymptotic 95% Confidence Interval: Upper Bound
0.560	0.058	0.348	0.446	0.674

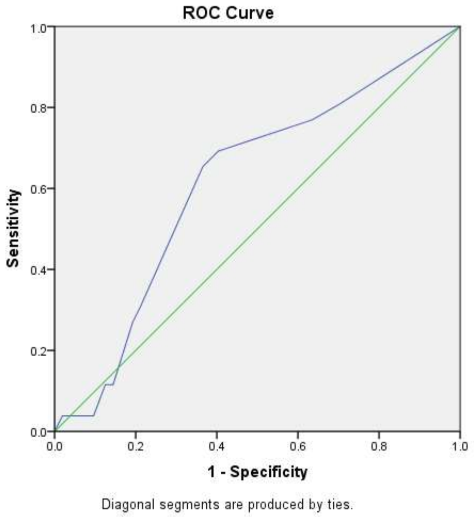

Figure S1b. ROC curve for CD8, AUC value is shown in the table below

Area Under the Curve

Test Result Variable(s): CD8

			Asymptotic 95% Confidence Interval	
Area	Std. Error[a]	Asymptotic Sig.[b]	Lower Bound	Upper Bound
0.614	0.061	0.074	0.495	0.732

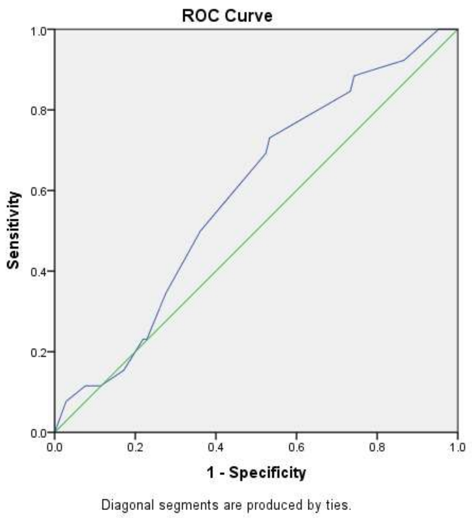

Figure S1c. ROC curve for CD45RO, AUC value is shown in the table below

Area Under the Curve

Test Result Variable(s): CD45RO

Area	Std. Error[a]	Asymptotic Sig.[b]	Asymptotic 95% Confidence Interval	
			Lower Bound	Upper Bound
0.590	0.059	0.155	0.475	0.705

Table S1. Univariate Cox logistic regression analysis for individual clinical variables

**Variables**	**HR (95%CI)** (n=264*)	**P**
Age (years)	1.632 (0.77-3.47)	0.203
T-stage	1.752 (1.00-3.06)	**0.049**
Histology	0.263 (0.06-1.08)	0.064
Grade	2.621 (1.50-4.60)	**0.001**
N stage	2.212 (1.27-3.85)	**0.005**
LVI	2.105 (1.10-4.02)	0.024
HR status[$]	2.726 (1.43-5.21)	**0.002**
HER2 status	2.708 (1.46-5.01)	**0.002**
p53	0.814 (0.46-1.46)	0.490
bcl2	0.520 (0.30-0.91)	0.022
Ki-67[&]	3.617 (2.05-6.39)	**<0.001**
Tumor subtypes		
Luminal A	1	
Luminal B	2.311 (1.10-4.86)	**0.027**
HER2 positive	4.380 (2.11-9.08)	**<0.001**
Triple-negative	3.955 (1.73-9.04)	**0.001**

*Only patients without missing survival data were included

Table S2. Univariate Cox logistic regression analysis for individual studied TILs and PFS, cut-off values were defined based on the results of AUC analysis

							95.0% CI for Exp(B)	
	**B**	**SE**	**Wald**	**df**	**Sig.**	**Exp(B)**	**Lower**	**Upper**
CD3>6%	0.169	0.294	0.331	1	0.565	1.184	0.666	2.105
CD8>6%	0.606	0.295	4.214	1	**0.040**	1.833	1.028	3.270
CD45>12.5%	0.769	0.336	5.247	1	**0.022**	2.159	1.117	4.169

Table S3. Univariate Cox logistic regression analysis for studied TILs, stratified by clinicopathological categories and PFS

Variables	Categories	N (n=282)	CD3^{high} HR (95%CI) (n=242)	P	CD8^{high} HR (95%CI) (n=261)	P	CD45RO^{high} HR (95%CI) (n=265)	P
Age (years)	≤ 50	67	0.79 (0.20-3.15)	0.73	1.98 (0.47-8.30)	0.35	2.00 (0.40-9.93)	0.40
	> 50	214	1.29 (0.68-2.42)	0.44	1.83 (0.97-0.44)	0.06	2.23 (1.08-0.58)	**0.03**
T-stage	T1	186	1.20 (0.55-2.59)	0.65	2.36 (1.09-5.12)	**0.03**	2.81 (1.13-7.01)	**0.03**
	T2 and more	85	1.08 (0.46-2.57)	0.86	1.20 (0.50-2.90)	0.68	1.39 (0.53-3.61)	0.51
Histology	IDC	237	1.10 (0.61-1.98)	0.76	1.74 (0.96-3.14)	0.07	2.18 (1.10-4.31)	**0.03**
	Others	36	1.61 (0.10-25.89)	0.73	1.85 (0.12-29.79)	0.66	1.03 (0.06-16.55)	0.98
Grade	Low and intermediate	174	0.94 (0.38-2.29)	0.89	2.63 (1.09-6.35)	**0.03**	1.78 (0.70-4.52)	0.23
	High	99	0.89 (0.39-2.08)	0.80	0.13 (1.00-0.71)	0.71	2.17 (0.75-6.30)	0.15
N stage	N0	185	0.76 (0.32-1.80)	0.53	1.95 (0.84-4.50)	0.12	1.90 (0.77-4.66)	0.16
	N+	94	1.53 (0.67-3.46)	0.31	1.58 (0.70-3.56)	0.27	1.99 (0.74-5.38)	0.17
LVI	Absent	177	1.02 (0.45-2.32)	0.96	2.12 (0.92-4.89)	0.08	1.87 (0.76-4.58)	0.17
	Present	103	1.24 (0.38-4.02)	0.73	1.72 (0.54-5.50)	0.36	1.97 (0.54-7.18)	0.30
HR status$	Negative	40	0.55 (0.16-1.90)	0.35	0.56 (0.16-1.93)	0.36	1.20 (0.26-5.59)	0.82
	Positive	241	1.21 (0.63-2.33)	0.57	2.04 (1.06-3.94)	**0.03**	2.13 (1.02-4.43)	**0.04**
HER2 status	Negative	238	1.36 (0.69-2.68)	0.37	1.82 (0.94-3.53)	0.08	2.07 (0.99-4.32)	0.05
	Amplified	43	0.49 (0.16-1.46)	0.20	1.30 (0.39-4.32)	0.67	1.47 (0.32-6.69)	0.62
p53	Negative	177	1.57 (0.76-3.25)	0.23	2.07 (1.01-4.22)	**0.05**	3.20 (1.30-7.86)	**0.01**
	Positive	103	0.75 (0.29-1.94)	0.55	1.50 (0.56-4.07)	0.42	1.21 (0.45-3.28)	0.71
bcl2	Negative	79	0.94 (0.38-2.34)	0.90	1.01 (0.41-2.51)	0.98	2.01 (0.67-6.06)	0.22
	Positive	202	1.24 (0.59-2.60)	0.57	2.37 (1.12-5.01)	**0.03**	2.04 (0.89-4.66)	0.09
Ki-67&	Low	180	0.98 (0.37-2.56)	0.96	1.62 (0.66-3.98)	0.30	1.73 (0.67-4.47)	0.26
	High	101	0.71 (0.34-1.51)	0.38	1.15 (0.53-2.54)	0.72	1.46 (0.55-3.84)	0.45
Tumor subtypes	Luminal A	148	1.66 (0.56-4.93)	0.36	2.69 (0.96-7.55)	0.06	1.99 (0.67-5.95)	0.22
	Luminal B	57	0.80 (0.25-2.49)	0.70	1.02 (0.33-3.16)	0.97	1.63 (0.43-6.15)	0.47
	HER2 positive	43	0.49 (0.16-1.46)	0.20	1.30 (0.39-4.32)	0.67	1.47 (0.32-6.69)	0.62
	Triple-negative	33	0.69 (0.17-2.77)	0.60	0.61 (0.15-2.43)	0.48	1.15 (0.24-5.59)	0.86

The total number of samples analyzed in the study was n = 282; only cases with valid information on individual variables were included in the table; $ negative for both or positive for either with cut-off 1%; &cut-off 20%; Abbreviations: IDC, Invasive ductal carcinoma; LVI, Lymphovascular invasion; HR, Hormonal receptor

Table S4. qRT-PCR results for control samples

nb	*GAPDH* (Ct)	*SNAI1* (Ct)	*SNAI1* relative expression	*SNAI2* (Ct)	*SNAI2* relative expression	*TWIST1* (Ct)	*TWIST1* relative expression	*ZEB1* (Ct)	*ZEB1* relative expression
1	23.11			0	0.0000	37.59	0.0000		
2	25.92	35.63	0.0012	0.00	0.0000			30.86	0.0327
3	28.64			0.00	0.0000	0.00	0.0000	33.31	0.0394
4	28.21	38.43	0.0008	0.00	0.0000	0	0.0000	32.80	0.0415
5	23.07	29.325	0.0131	0	0.0000			30.59	0.0054
6	22.91	31.605	0.0024			35.795	0.0001	29.955	0.0076
7	24.90			0	0.0000	0	0.0000		
8	21.84	30.38	0.0027	0.00	0.0000	36.51	0.0000	29.07	0.0067
9	26.455	37.41	0.0005	0	0.0000	0	0.0000	32.08	0.0203
10	23.945	31.595	0.0050	0	0.0000	38.43	0.0000	31.47	0.0054
11	28.20	35.62	0.0059	0.00	0.0000	0.00	0.0000	31.61	0.0941
12	27.24	34.68	0.0058	0.00	0.0000	0.00	0.0000	30.65	0.0944
13	24.575	32.525	0.0040	0	0.0000			31.07	0.0111
14	23.465	31.47	0.0039	0	0.0000			30.905	0.0058
15	27.12	33.68	0.0106	0.00	0.0000			30.96	0.0696
16	26.42	34.76	0.0031	0.00	0.0000	0.00	0.0000	30.93	0.0439
17	23	31.225	0.0033	0	0.0000			29.905	0.0083
18	23.63			0	0.0000	38.23	0.0000		
19	24.7	30.915	0.0135	0	0.0000	0	0.0000	30.37	0.0196
20	27.685	34.22	0.0108	0	0.0000	0	0.0000	31.285	0.0825
21	23.745	33.83	0.0009	0	0.0000	41.36	0.0000	30.98	0.0066
22	23.48	33.59	0.0009	0.00	0.0000	34.11	0.0006	31.52	0.0038
23	25.91	33.98	0.0037	0.00	0.0000	0.00	0.0000	30.60	0.0387
24	24.665	32.87	0.0034	0	0.0000			30.16	0.0222
25	23.735	32.715	0.0020	0	0.0000	0	0.0000	30.42	0.0097
26	27.43	35.14	0.0048	0.00	0.0000	37.81	0.0008	32.05	0.0408
27	24.43	33.07	0.0025	0.00	0.0000			30.12	0.0194
28	27.065	33.015	0.0162	0	0.0000	0	0.0000	31.195	0.0571
29	21.91	31.06	0.0018	0	0.0000	0	0.0000	30.23	0.0031
30	22.79			0	0.0000	35.96	0.0001		
31	22.57			0	0.0000	36.61	0.0001		
32	24.94			0	0.0000			29.44	0.0442
33	29.065	38.12	0.0019	0	0.0000	0	0.0000	33.32	0.0524
34	23.395	32.525	0.0018	0	0.0000	0	0.0000	29.675	0.0129
35	25.00	32.34	0.0062	0.00	0.0000			30.06	0.0300
36	23.035	31.77	0.0023	0	0.0000	0	0.0000	30.84	0.0045
37	27.06	35.41	0.0031	0.00	0.0000	0.00	0.0000	31.46	0.0472
38	29.35	36.00	0.0100	0.00	0.0000	0.00	0.0000	31.93	0.1678
39	26.61			0	0.0000	0	0.0000		
40	28.68			0.00	0.0000	0	0.0000	36.19	0.0055
41	24.30	33.65	0.0015	0.00	0.0000	0.00	0.0000	30.88	0.0105
42	27.61	35.53	0.0041	0.00	0.0000	0	0.0000	32.58	0.0319
43	23.41			0	0.0000	37.29	0.0001		
44	22.765	33.4	0.0006	0	0.0000	0	0.0000	31.305	0.0027

45	22.53	31.925	0.0015	0	0.0000	0	0.0000	30.42	0.0042
46	23.61	33.335	0.0012	0	0.0000	0	0.0000	29.63	0.0154
47	23.91	30.505	0.0103	0	0.0000	0	0.0000	30.515	0.0103
48	23.99			0	0.0000	0	0.0000		
49	22.02	31.325	0.0016	0	0.0000			30.83	0.0022
50	24.21			0	0.0000	36.18	0.0003		
51	24.72	35.10	0.0008	0.00	0.0000	0	0.0000	30.17	0.0229
52	23.38			0	0.0000	0	0.0000		
53	26.745	31.45	0.0383	0	0.0000			30.85	0.0581
54	21.615	29.375	0.0046			38.24	0.0000	29.525	0.0042
55	21.805	31.13	0.0016	0	0.0000			30.605	0.0022
56	21.975	32.16	0.0009			0	0.0000	29.955	0.0040
57	23.80	32.09	0.0032	0.00	0.0000	0.00	0.0000	30.35	0.0106
58	22.735	30.095	0.0061	0	0.0000			30.695	0.0040
59	27.43	34.67	0.0066	0	0.0000	0	0.0000	30.82	0.0954
60	24.57	32.92	0.0031	0	0.0000			31.62	0.0075

Table S5. qRT-PCR results for patient samples

nb	GAPDH (Ct)	*SNAI1* (Ct)	*SNAI1* relative expression	CTC EMT	*SNAI2* (Ct)	*SNAI2* relative expression	CTC EMT	*TWIST1* (Ct)	*TWIST1* relative expression	CTC EMT	*ZEB1* (Ct)	*ZEB1* relative expression	CTC EMT
1	26.05	33.65	0.0051	Fail	0.00	0.000000	Fail	0	0.000000	Fail	31.28	0.026645	Fail
2	23.70	34.04	0.0008	Fail	0.00	0.000000	Fail	35.80	0.000228	Fail	31.71	0.003879	Fail
3	23.68	34.13	0.0007	Fail	0	0.000000	Fail	37.685	0.000061	Fail	31.685	0.003893	Fail
4	22.43				0	0.000000	Fail	36.17	0.000073	Fail			
5	23.18	31.86	0.0024	Fail	0.00	0.000000	Fail				32.68	0.001381	Fail
6	23.62				0	0.000000	Fail	39.48	0.000017	Fail			
7	21.69	29.18	0.0056	Fail	0	0.000000	Fail	0	0.000000	Fail	31.025	0.001548	Fail
8	26.34	34.905	0.0026	Fail	0	0.000000	Fail				31.19	0.034674	Fail
9	26.03				0	0.000000	Fail	38.51	0.000175	Fail			
10	22.81				0	0.000000	Fail	36.00	0.000107	Fail			
11	22.77				0	0.000000	Fail	36.51	0.000073	Fail			
12	25.22	35.60	0.0008	Fail	0.00	0.000000	Fail	0	0.000000	Fail	33.17	0.004058	Fail
13	25.24	34.84	0.0013	Fail	0.00	0.000000	Fail	0	0.000000	Fail	30.16	0.032918	Fail
14	22.32				0	0.000000	Fail	35.12	0.000139	Fail			
15	23.67				0	0.000000	Fail	37.34	0.000077	Fail			
16	23.56	31.25	0.0048	Fail	0.00	0.000000	Fail	0.00	0.000000	Fail	32.95	0.001490	Fail
17	24.25				0	0.000000	Fail	37.59	0.000097	Fail			
18	21.38				39.91	0.000003	**Pass**	33.93	0.000167	Fail			
19	26.64	34.495	0.0043	Fail	0	0.000000	Fail	0	0.000000	Fail	32.265	0.020263	Fail
20													
21	21.33				38.95	0.000005	**Pass**	33.99	0.000154	Fail			
22	22.40				38.91	0.000011	**Pass**	36.09	0.000076	Fail			
23	23.36				0	0.000000	Fail						
24	22.46				0	0.000000	Fail	36.28	0.000069	Fail			
25	24.50				0	0.000000	Fail	36.04	0.000335	Fail			
26	26.69				0	0.000000	Fail	0	0.000000	Fail			
27	24.93				0	0.000000	Fail	36.28	0.000382	Fail			
28	23.58	33.85	0.0008	Fail	0.00	0.000000	Fail	35.45	0.000266	Fail	30.46	0.008461	Fail

29	22.98	31.46	0.0028	Fail	0.00	0.000000	Fail	34.32	0.000384	Fail	29.36	0.012007	Fail
30	24.21				40.87	0.000010	**Pass**	37.87	0.000077	Fail			
31	22.605	31.395	0.0023	Fail	0	0.000000	Fail				30.81	0.003389	Fail
32	22.76				0	0.000000	Fail	0	0.000000	Fail			
33	23.38				0	0.000000	Fail	37.81	0.000045	Fail			
34	23.41	32.53	0.0018	Fail	0	0.000000	Fail				33.905	0.000693	Fail
35	23.79				0	0.000000	Fail	0	0.000000	Fail			
36	27.075	34.215	0.0071	Fail	0	0.000000	Fail	0	0.000000	Fail	31.27	0.054598	Fail
37	23.19				0	0.000000	Fail	36.58	0.000093	Fail			
38	22.65				0	0.000000	Fail	36.76	0.000056	Fail			
39	22.425	31.835	0.0015	Fail	39.84	0.000006	**Pass**				29.865	0.005759	Fail
40	23.80				0	0.000000	Fail						
41													
42	21.96				0	0.000000	Fail	35.08	0.000112	Fail			
43	26.215	34.765	0.0027	Fail				0	0.000000	Fail	29.945	0.075363	Fail
44	22.65				38.81	0.000014	**Pass**	35.66	0.000121	Fail			
45	23.32				0	0.000000	Fail	0	0.000000	Fail			
46	22.89				41.71	0.000002	**Pass**	35.01	0.000224	Fail			
47	21.24	29.03	0.0045	Fail	0	0.000000	Fail	37.05	0.000017	Fail	29.535	0.003184	Fail
48	25.03				41.15	0.000014	**Pass**	36.05	0.000479	Fail			
49	26.85				0	0.000000	Fail	0	0.000000	Fail			
50	29.61				0	0.000000	Fail	0	0.000000	Fail			
51	23.24				0	0.000000	Fail	36.86	0.000080	Fail			
52	21.99				0	0.000000	Fail	38.66	0.000010	Fail			
53	22.43				0	0.000000	Fail	0	0.000000	Fail			
54	23.20				0	0.000000	Fail	35.56	0.000191	Fail			
55	22.64				0	0.000000	Fail	39.05	0.000011	Fail			
56	24.57	33.56	0.0020	Fail	0	0.000000	Fail				34.215	0.001249	Fail
57	23.04				0	0.000000	Fail	37.05	0.000061	Fail			
58	24.04				0	0.000000	Fail	37.12	0.000116	Fail			
59	23.71				0	0.000000	Fail	37.00	0.000100	Fail			

60	22.42				0	0.000000	Fail	35.79	0.000094	Fail			
61	23.97				0	0.000000	Fail	0	0.000000	Fail			
62													
63	24.17				0	0.000000	Fail	37.57	0.000093	Fail			
64	22.70				0	0.000000	Fail	36.76	0.000058	Fail			
65	21.31				40.32	0.000002	**Pass**						
66	23.75				0	0.000000	Fail	0	0.000000	Fail			
67	23.47				0	0.000000	Fail	36.28	0.000139	Fail			
68	27.53				0	0.000000	Fail	0	0.000000	Fail			
69	25.21				0	0.000000	Fail	39.12	0.000065	Fail			
70	25.31				0	0.000000	Fail	38.55	0.000103	Fail			
71	24.47	34.37	0.0011	Fail	0.00	0.000000	Fail	0	0.000000	Fail	30.85	0.012007	Fail
72	25.05				0	0.000000	Fail	36.59	0.000337	Fail			
73	26.995	34.105	0.0072	Fail	0	0.000000	Fail				30.965	0.063813	Fail
74	23.225	31.96	0.0023	Fail	0	0.000000	Fail	35.635	0.000184	Fail	30.555	0.006215	Fail
75	23.33				0	0.000000	Fail	37.00	0.000077	Fail			
76	29.68				0	0.000000	Fail	0	0.000000	Fail			
77	22.49				39.92	0.000006	**Pass**	36.14	0.000078	Fail			
78	23.765	30.345	0.0105	Fail	0	0.000000	Fail				30.65	0.008461	Fail
79	22.08				35.39	0.000098	**Pass**	38.18	0.000014	Fail			
80	20.68				37.81	0.000007	**Pass**	36.34	0.000019	Fail			
81	24.07				0	0.000000	Fail	35.84	0.000285	Fail			
82	21.83				40.16	0.000003	**Pass**	37.19	0.000024	Fail			
83	24.01				37.91	0.000066	**Pass**	37.23	0.000105	Fail			
84	21.995	30.635	0.0025	Fail	0	0.000000	Fail	37.975	0.000015	Fail	31.315	0.001565	Fail
85	25.39				0	0.000000	Fail						
86	23.51				38.92	0.000023	**Pass**	37.87	0.000048	Fail			
87													
88	23.32				0	0.000000	Fail	37.71	0.000047	Fail			
89	21.64				0	0.000000	Fail	35.84	0.000053	Fail			
90	23.38				0	0.000000	Fail	35.68	0.000198	Fail			

91	23.12				0	0.000000	Fail	37.22	0.000057	Fail			
92	24.94	35.76	0.0006	Fail	0.00	0.000000	Fail	0	0.000000	Fail	34.63	0.001215	Fail
93	23.23				0	0.000000	Fail	38.55	0.000024	Fail			
94	23.50				38.59	0.000029	**Pass**	37.61	0.000056	Fail			
95	24.16				0	0.000000	Fail	38.52	0.000048	Fail			
96	22.67				0	0.000000	Fail	37.53	0.000034	Fail			
97	23.40	31.06	0.0050	Fail	0.00	0.000000	Fail	0.00	0.000000	Fail	29.21	0.017824	Fail
98	23.03				39.27	0.000013	**Pass**	35.34	0.000196	Fail			
99	21.19				0	0.000000	Fail	37.50	0.000012	Fail			
100	23.94				0	0.000000	Fail	0	0.000000	Fail			
101	22.64				0	0.000000	Fail	37.83	0.000027	Fail			
102	22.60				0	0.000000	Fail	36.65	0.000059	Fail			
103	24.69				0	0.000000	Fail	0	0.000000	Fail			
104	22.74				0	0.000000	Fail	37.42	0.000038	Fail			
105	25.14	33.89	0.0023	Fail	0	0.000000	Fail	0	0.000000	Fail	33.37	0.003331	Fail
106	21.58				0	0.000000	Fail	36.79	0.000026	Fail			
107	23.51				0	0.000000	Fail	38.21	0.000038	Fail			
108	25.41	33.42	0.0039	Fail	0.00	0.000000	Fail	34.83	0.001455	**Pass**	30.22	0.035649	Fail
109	24.72	32.495	0.0046	Fail	0	0.000000	Fail				32.595	0.004260	Fail
110	26.85				0	0.000000	Fail	38.22	0.000377	Fail			
111	23.49				0	0.000000	Fail	0	0.000000	Fail			
112	25.87	32.96	0.0073	Fail	0.00	0.000000	Fail	0	0.000000	Fail	30.54	0.039418	Fail
113	25.72				0	0.000000	Fail	39.05	0.000097	Fail			
114	22.105	31.065	0.0020	Fail	0	0.000000	Fail	36.285	0.000054	Fail	30.51	0.002950	Fail
115	22.67				0	0.000000	Fail	37.97	0.000025	Fail			
116	22.33				0	0.000000	Fail	35.37	0.000118	Fail			
117	22.55				37.97	0.000023	**Pass**	0	0.000000	Fail			
118	22.84				40.81	0.000004	**Pass**	37.05	0.000053	Fail			
119	24.98				0	0.000000	Fail	0	0.000000	Fail			
120	25.87				0	0.000000	Fail	0	0.000000	Fail			
121	26.715	36.97	0.0008	Fail	0	0.000000	Fail	0	0.000000	Fail	34.685	0.003988	Fail

122	29.70				0	0.000000	Fail	38.72	0.001926	**Pass**			
123	26.33				0	0.000000	Fail	0	0.000000	Fail			
124	25.58	34.44	0.0021	Fail	0.00	0.000000	Fail	0	0.000000	Fail	30.73	0.028067	Fail
125	22.61				0	0.000000	Fail	36.27	0.000077	Fail			
126	23.62				38.84	0.000026	**Pass**	37.45	0.000069	Fail			
127	25.14	33.93	0.0023	Fail	40.69	0.000021	**Pass**	36.26	0.000451	Fail	30.96	0.017701	Fail
128	23.72				0	0.000000	Fail	37.58	0.000067	Fail			
129	23.69	33.56	0.0011	Fail	0.00	0.000000	Fail				32.03	0.003086	Fail
130	25.81				0	0.000000	Fail	40.18	0.000047	Fail			
131	26.97				0	0.000000	Fail	0	0.000000	Fail			
132	26.08				0	0.000000	Fail						
133	26.62	33.71	0.0073	Fail	0	0.000000	Fail				29.865	0.105477	Fail
134	22.74				39.26	0.000011	**Pass**	37.53	0.000035	Fail			
135	21.15				0	0.000000	Fail	34.65	0.000086	Fail			
136	22.92				0	0.000000	Fail	36.11	0.000106	Fail			
137	23.18				0	0.000000	Fail	34.16	0.000496	Fail			
138	23.65				0	0.000000	Fail	37.05	0.000092	Fail			
139	21.88	30.67	0.0023	Fail	0.00	0.000000	Fail				28.01	0.014229	Fail
140													
141	22.34				0	0.000000	Fail	36.35	0.000060	Fail			
142	22.71				39.82	0.000007	**Pass**	35.53	0.000138	Fail			
143	25.23				0	0.000000	Fail	37.88	0.000155	Fail			
144	22.78				37.82	0.000030	**Pass**	38.37	0.000020	Fail			
145	23.03				40.17	0.000007	**Pass**						
146	24.66				0.00	0.000000	Fail	0	0.000000	Fail			
147	22.99				0	0.000000	Fail	37.65	0.000039	Fail			
148	24.25				0	0.000000	Fail						
149	23.01				0	0.000000	Fail	37.67	0.000039	Fail			
150	22.57				0	0.000000	Fail	0.00	0.000000	Fail			
151	23.68				38.31	0.000039	**Pass**	38.53	0.000034	Fail			
152	22.99				39.02	0.000015	**Pass**	36.49	0.000086	Fail			

153	24.12				0	0.000000	Fail	37.83	0.000074	Fail			
154	22.23	31.455	0.0017	Fail	0	0.000000	Fail				29.925	0.004826	Fail
155	22.71				41.36	0.000002	**Pass**	33.66	0.000503	Fail			
156	24.96	30.86	0.0168	Fail	0.00	0.000000	Fail	36.23	0.000405	Fail	30.20	0.026461	Fail
157	22.63				0	0.000000	Fail						
158	25.01				0	0.000000	Fail						
159	22.37				0	0.000000	Fail	38.50	0.000014	Fail			
160	28.65				0	0.000000	Fail	38.51	0.001080	**Pass**			
161	22.52				0	0.000000	Fail						
162	24.92				0	0.000000	Fail	37.03	0.000226	Fail			
163	27.37				0	0.000000	Fail	0	0.000000	Fail			
164	22.87				0	0.000000	Fail	37.02	0.000055	Fail			
165	22.92				37.37	0.000045	**Pass**	38.91	0.000015	Fail			
166	23.47	33.91	0.0007	Fail	0.00	0.000000	Fail				32.15	0.002438	Fail
167													
168	21.46				40.48	0.000002	**Pass**	34.85	0.000093	Fail			
169													
170	23.10	31.07	0.0040	Fail	40.60	0.000005	**Pass**	32.76	0.001232	**Pass**	31.21	0.003607	Fail
171	23.27	32.37	0.0018	Fail	0.00	0.000000	Fail				29.12	0.017397	Fail
172	23.75	32.36	0.0026	Fail	0.00	0.000000	Fail	36.93	0.000108	Fail	28.87	0.028756	Fail
173	23.18				38.38	0.000027	**Pass**	36.51	0.000097	Fail			
174	22.39	31.595	0.0017	Fail	0	0.000000	Fail	40.595	0.000003	Fail	30.47	0.003696	Fail
175	22.90				0	0.000000	Fail	37.42	0.000042	Fail			
176	20.94				37.36	0.000011	**Pass**	35.67	0.000037	Fail			
177	27.38				0	0.000000	Fail	0	0.000000	Fail			
178	23.61				0	0.000000	Fail	36.32	0.000149	Fail			
179	26.72				0	0.000000	Fail	0	0.000000	Fail			
180	23.37				0	0.000000	Fail	38.22	0.000034	Fail			
181	21.62				0	0.000000	Fail	36.07	0.000045	Fail			
182	21.45				38.76	0.000006	**Pass**	36.79	0.000024	Fail			
183	22.25				38.91	0.000010	**Pass**	34.71	0.000177	Fail			

184	24.79	34.31	0.0014	Fail	0.00	0.000000	Fail				34.07	0.001614	Fail
185	23.33				40.45	0.000007	**Pass**						
186	23.97				39.00	0.000030	**Pass**	0	0.000000	Fail			
187	23.08	29.765	0.0097	Fail	0	0.000000	Fail	0	0.000000	Fail	31.42	0.003086	Fail
188													
189	29.53				0	0.000000	Fail	0	0.000000	Fail			
190	23.89				0	0.000000	Fail	0	0.000000	Fail			
191	24.74				40.59	0.000017	**Pass**	40.16	0.000023	Fail			
192	23.23				0	0.000000	Fail						
193	27.735	35.465	0.0047	Fail	0	0.000000	Fail	0	0.000000	Fail	33.75	0.015463	Fail
194	23.69				0	0.000000	Fail	36.90	0.000105	Fail			
195													
196	27.49				0	0.000000	Fail	0	0.000000	Fail			
197	26.39				0	0.000000	Fail	0	0.000000	Fail			
198	22.20				0	0.000000	Fail						
199	23.76				0	0.000000	Fail	38.12	0.000047	Fail			
200	24.275	31.85	0.0052	Fail	0	0.000000	Fail				29.375	0.029157	Fail
201	23.18				0	0.000000	Fail	37.17	0.000061	Fail			
202	22.27				0	0.000000	Fail	36.78	0.000043	Fail			
203	28.76				0	0.000000	Fail	0	0.000000	Fail			
204	24.42				0	0.000000	Fail	39.30	0.000033	Fail			
205	23.50				0	0.000000	Fail	37.40	0.000065	Fail			
206	23.81	32.345	0.0027	Fail	0	0.000000	Fail	0	0.000000	Fail	31.25	0.005759	Fail
207	24.97	36.97	0.0002	Fail	0.00	0.000000	Fail				34.12	0.001760	Fail
208	21.86	30.325	0.0028	Fail	42	0.000001	**Pass**	0	0.000000	Fail	30.21	0.003065	Fail
209	22.74				0	0.000000	Fail	36.70	0.000062	Fail			
210	24.44				0	0.000000	Fail	0	0.000000	Fail			
211	23.65				0	0.000000	Fail	34.59	0.000509	Fail			
212	22.43				0	0.000000	Fail	38.10	0.000019	Fail			
213	24.58				0	0.000000	Fail	0	0.000000	Fail			
214	21.99				0	0.000000	Fail	37.41	0.000023	Fail			

215	26.50				0	0.000000	Fail	37.52	0.000482	Fail			
216	21.90				0	0.000000	Fail	35.43	0.000084	Fail			
217	25.07	34.13	0.0019	Fail	0.00	0.000000	Fail	0	0.000000	Fail	30.75	0.019438	Fail
218	22.56				0	0.000000	Fail						
219	28.06	34.70	0.0100	Fail	0.00	0.000000	Fail	0	0.000000	Fail	31.96	0.066754	Fail
220	23.35				0	0.000000	Fail	37.44	0.000057	Fail			
221	22.22				0	0.000000	Fail	37.08	0.000034	Fail			
222													
223	23.49				0	0.000000	Fail	36.97	0.000087	Fail			
224	22.84				0	0.000000	Fail	37.25	0.000046	Fail			
225	23.81				0	0.000000	Fail	0	0.000000	Fail			
226													
227	28.13				0	0.000000	Fail	0	0.000000	Fail			
228	24.10				0	0.000000	Fail	39.85	0.000018	Fail			
229	22.485	32.05	0.0013	Fail	0	0.000000	Fail	37.51	0.000030	Fail	31.24	0.002315	Fail
230	23.79				0	0.000000	Fail	36.36	0.000164	Fail			
231	23.77	32.095	0.0031	Fail	0	0.000000	Fail				30.515	0.009323	Fail
232	23.57				0	0.000000	Fail	37.61	0.000059	Fail			
233	23.05				0	0.000000	Fail	36.27	0.000105	Fail			
234													
235													
236													
237	24.65				0	0.000000	Fail	36.84	0.000214	Fail			
238	23.39				0	0.000000	Fail	36.08	0.000151	Fail			
239	23.26				0	0.000000	Fail	0	0.000000	Fail			
240	21.79				0	0.000000	Fail	34.08	0.000200	Fail			
241	27.17				0	0.000000	Fail	0	0.000000	Fail			
242	25.24				0	0.000000	Fail	0	0.000000	Fail			
243	21.16				0	0.000000	Fail						
244	22.53				0	0.000000	Fail	38.10	0.000021	Fail			
245	23.63				40.31	0.000010	**Pass**						

246	24.13				0	0.000000	Fail	38.25	0.000056	Fail			
247	25.58	36.09	0.0007	Fail	0.00	0.000000	Fail	0	0.000000	Fail	32.63	0.007546	Fail
248	23.03				0	0.000000	Fail	37.66	0.000040	Fail			
249	24.55	32.195	0.0050	Fail	0	0.000000	Fail				29.865	0.025120	Fail
250	24.90				0	0.000000	Fail	0	0.000000	Fail			
251	22.61				0	0.000000	Fail	34.66	0.000235	Fail			
252	22.93	31.60	0.0024	Fail	40.02	0.000007	**Pass**				28.93	0.015571	Fail
253	23.76				0	0.000000	Fail	37.74	0.000062	Fail			
254	23.65				0	0.000000	Fail						
255	28.26				0	0.000000	Fail	0	0.000000	Fail			
256	27.37				0	0.000000	Fail						
257	22.17	31.58	0.0015	Fail	0	0.000000	Fail	36.795	0.000040	Fail	31.57	0.001480	Fail
258	22.53				40.98	0.000003	**Pass**	37.80	0.000025	Fail			
259	22.60				0	0.000000	Fail	36.92	0.000049	Fail			
260	24.47				0	0.000000	Fail	0	0.000000	Fail			
261	23.28				0	0.000000	Fail	37.23	0.000063	Fail			
262	25.77				0	0.000000	Fail	0	0.000000	Fail			
263	23.645	32.905	0.0016	Fail	37.45	0.000070	**Pass**	38.925	0.000025	Fail	30.975	0.006215	Fail
264	22.71				0	0.000000	Fail	36.84	0.000056	Fail			
265	29.02				0	0.000000	Fail	0	0.000000	Fail			
266	22.285	31.44	0.0018	Fail				36.245	0.000063	Fail	29.945	0.004944	Fail
267													
268	25.53				0	0.000000	Fail	0	0.000000	Fail			
269	25.54				0	0.000000	Fail	37.31	0.000285	Fail			
270	25.58	33.64	0.0037	Fail	0.00	0.000000	Fail	0	0.000000	Fail	29.68	0.058517	Fail
271	24.46				0	0.000000	Fail	38.73	0.000051	Fail			
272	27.06				0.00	0.000000	Fail	0	0.000000	Fail	31.92	0.034554	Fail
273	23.40				0	0.000000	Fail	0	0.000000	Fail			
274	23.12				0	0.000000	Fail	0	0.000000	Fail			
275	23.03				38.50	0.000022	**Pass**	36.70	0.000077	Fail			
276	24.91				0	0.000000	Fail						

277	23.23				0	0.000000	Fail	0	0.000000	Fail			
278	22.53				0	0.000000	Fail	38.08	0.000021	Fail			
279	32.40				0	0.000000	Fail	0	0.000000	Fail			
280	23.42	31.67	0.0033	Fail	0.00	0.000000	Fail				28.85	0.023115	Fail
281	24.08				0	0.000000	Fail						
282	24.51				0	0.000000	Fail	35.72	0.000421	Fail			

Table S6. Measured cytokines median levels (pg/ml)

	Median	Min.	Max.	Range
TGF-β1	19144.41	3696.87	129499.59	125802.72
TGF-β2	2096.89	809.1	4190.22	3381.12
TGF-β3	662.13	35.55	1630.45	1594.9
IFN-alpha2	99.21	43.39	176.41	133.02
IL-1alpha	0.28	0.02	4.76	4.74
IL-2Ralpha	71.38	21.2	149.43	128.23
IL-3	38.18	1.35	145.37	144.02
IL-12(p40)	80.57	0.55	2260.55	2260
IL-16	337.91	147.57	1115.07	967.5
IL-18	44.9	0.83	677.64	676.81
CTACK	1321.14	289.08	3098.73	2809.65
GRO-alpha	3.11	3.11	12.93	9.82
HGF	275.77	81.34	6374.22	6292.88
LIF	6.75	0.01	27.15	27.14
MCP-3	52.49	0.57	197.19	196.62
M-CSF	9.4	0.2	52.26	52.06
MIF	275.5	73.29	2710.28	2636.99
MIG	599.1	123.46	5497.58	5374.12
Beta-NGF	0.95	0.04	20.4	20.36
SCF	48.27	5.41	164.19	158.78
SCGF-beta	16763.06	4385.21	77107.19	72721.98
SDF-1alpha	219.53	18.7	637.26	618.56
TNF-beta	0.37	0.04	2.83	2.79
TRAIL	40.52	3.63	154.5	150.87
IL-1beta	4.51	1.13	23.48	22.35
Il-1r alpha	224.81	41.12	13299.4	13258.28
IL-2	27.89	1.91	1167.73	1165.82
IL-4	7.47	1.76	29.95	28.19
IL-5	8.96	2.29	39.91	37.62
IL-6	18.63	2.32	935.24	932.92
IL-7	11.9	1.58	144.64	143.06
IL-8	32.98	5.49	268.96	263.47
IL-9	21.24	4.66	136.04	131.38
IL-10	7.28	1.53	578.6	577.07
IL-12	24.4	0.94	1242.02	1241.08
IL-13	9.13	1.35	618.78	617.43
IL-15	17.09	1.43	81.2	79.77
IL-17	71.15	7.54	625.09	617.55
Eotaxin	420.99	38.73	1764.37	1725.64
FGF	132.38	38.5	603.48	564.98
G-CSF	46.71	2.47	296.96	294.49
GM-CSF	59.2	15.92	317.88	301.96
IFN-gamma	700.25	154.13	4180.03	4025.9
IP-10	2775.31	944.55	16562.03	15617.48
MCP-1	35.37	10.84	126.49	115.65
MIP-1alpha	6.44	2.88	27.1	24.22
MIP-1beta	67.9	24.86	677.76	652.9
PDGF-BB	1023.85	25.77	9310.17	9284.4
RANTES	6981.32	1184.95	29425.26	28240.31
TNF-alpha	93.76	26.42	562.5	536.08
VEGF	49.68	0.95	290.39	289.44
